# Asymmetric Network Combining CNN and Transformer for Building Extraction from Remote Sensing Images

**DOI:** 10.3390/s24196198

**Published:** 2024-09-25

**Authors:** Junhao Chang, Yuefeng Cen, Gang Cen

**Affiliations:** School of Information and Electronic Engineering, Zhejiang University of Science and Technology, Hangzhou 310023, China; cjh@zust.edu.cn (J.C.); gcen@163.com (G.C.)

**Keywords:** building extraction, bilateral hybrid attention, convolutional neural network, remote sensing, semantic segmentation, transformer

## Abstract

The accurate extraction of buildings from remote sensing images is crucial in fields such as 3D urban planning, disaster detection, and military reconnaissance. In recent years, models based on Transformer have performed well in global information processing and contextual relationship modeling, but suffer from high computational costs and insufficient ability to capture local information. In contrast, convolutional neural networks (CNNs) are very effective in extracting local features, but have a limited ability to process global information. In this paper, an asymmetric network (CTANet), which combines the advantages of CNN and Transformer, is proposed to achieve efficient extraction of buildings. Specifically, CTANet employs ConvNeXt as an encoder to extract features and combines it with an efficient bilateral hybrid attention transformer (BHAFormer) which is designed as a decoder. The BHAFormer establishes global dependencies from both texture edge features and background information perspectives to extract buildings more accurately while maintaining a low computational cost. Additionally, the multiscale mixed attention mechanism module (MSM-AMM) is introduced to learn the multiscale semantic information and channel representations of the encoder features to reduce noise interference and compensate for the loss of information in the downsampling process. Experimental results show that the proposed model achieves the best F1-score (86.7%, 95.74%, and 90.52%) and IoU (76.52%, 91.84%, and 82.68%) compared to other state-of-the-art methods on the Massachusetts building dataset, the WHU building dataset, and the Inria aerial image labeling dataset.

## 1. Introduction

With the rapid development of remote sensing technology, the accurate extraction of buildings from high-resolution remote sensing images is of great significance in the fields of 3D urban planning [1,2,3], disaster detection [4,5,6], and military reconnaissance [7,8]. This is crucial for practical applications, such as urban growth analysis, infrastructure development, and post-disaster damage assessment. Despite significant progress, the complexity of urban environments and the diversity of building appearances, as well as image blurring in high-resolution remotely sensed images, make complete and high-precision extraction of buildings from remotely sensed images still a challenging task.

Early building extraction methods mainly relied on manual interpretation and classical image processing techniques, such as edge detection, region growing, and morphological operations, to depict building boundaries. For example, Sobel and Canny edge detectors are widely used for building edge recognition, while region-based methods mainly classify and group pixels with similar features through segmentation algorithms. However, these methods are highly sensitive to noise interference and lighting conditions and have poor generalization ability, which prevents them from being widely used in various vision tasks. Another common traditional approach is the use of hand-designed features and rule-based systems, in which features such as texture, shape, color, and spectrum are hand-designed and used in combination to improve segmentation for building extraction. Although these methods have achieved some success, their hand-designed features have many shortcomings. For example, poor robustness in the face of complex backgrounds and easily interfered with by noise and shadows; inconsistent extraction results in the face of scale changes and different angles of buildings; reliance on low-level features; and lack of high-level semantic information, all of which affect the building extraction effect, and cannot be generalized to different datasets and various complex urban scenarios. Advances in aerospace technology and electronic sensor technology have made high-resolution remote sensing image data more abundant [9,10,11], laying a foundation for the development of building extraction methods based on deep learning.

**Figure 1:**
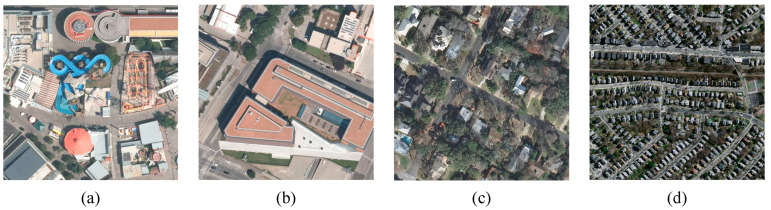
Challenges in the building extraction task: (**a**) Diversity of building forms. (**b**) Scale changes in buildings. (**c**) Interference from complex backgrounds. (**d**) Blurred remote sensing images.

In recent years, deep learning (DL) techniques have sparked a revolution in the fields of computer vision [12,13] and natural language processing [14], especially in image processing, which provides new possibilities for solving the building extraction problem for high-resolution remotely sensed imagery. This has transformed building extraction from traditional manual methods into more efficient automated methods. Deep learning-based building extraction methods can not only make reasonable use of the texture, color, and geometric information of buildings but also make use of building contextual spatial information. Compared with manual methods, deep learning methods are able to learn target features from multiple perspectives at the same time, which helps to further improve the accuracy of building extraction. Among them, convolutional neural networks (CNN) [15] and Transformer [16] have received considerable attention owing to their excellent performance, and are widely used in image semantic segmentation tasks. To fully utilize the advantages of CNN and Transformer, it is first necessary to reveal the problems that need to be faced when extracting buildings from remote sensing images. As shown in Figure 1, buildings have different shapes and styles, including circular, rectangular, and irregular shapes. This prevents the knowledge of a single shape style from being effectively applied to all buildings, increases the risk of misclassification through experience or rules, and increases the difficulty of edge detection, requiring more complex algorithms and models to capture diverse geometric features [17]. Second, buildings are subject to scale variations due to different sampling distances and viewing angles. For small-scale buildings, they may be masked or ignored by background noise; for large-scale buildings, they may be beyond the scope of a single remote sensing image block, resulting in feature loss [18]. In addition, complex backgrounds (e.g., vegetation, water bodies, shadows, and other interferences) may obscure the edges of buildings, increasing the difficulty of edge detection, while interferences in the background may have similar colors and textures as buildings, leading to misclassification [19]. Finally, remote sensing images may be blurred due to sensor technology limitations, atmospheric interference, and imaging motion trajectories during the acquisition process, making the edges and details of buildings blurred, making it difficult to distinguish between the buildings and the background, and further decreasing the accuracy of classification methods based on feature matching and edge detection [20]. In order to cope with the above difficulties in building extraction, we need to make reasonable use of deep learning methods to automatically learn local features such as the shape, color, and texture of the building, and at the same time, pay attention to the correlation between the target object and the information of the surrounding environment, so as to identify the influence of interfering objects on the overall shape features of the building, accurately locate the overall structure of the building, and further obtain good segmentation results.

CNN can automatically learn the detailed features of buildings through a large amount of labeled data without the need to manually design the features, which enables the model to be adapted to a variety of building types through training. CNN can effectively capture the local detailed features of a region, such as texture and edges, through convolutional operations. Meanwhile, the convolutional operations of multilayered convolutional kernels of different sizes can capture different levels of shape information from low to high, which enhances the model’s adaptability to buildings with diverse shapes [21]. However, due to the fixed size of the convolution kernel, CNN is insufficient for dealing with long-range dependencies and is prone to ignoring global information. Influenced by sampling distance and viewing angle factors, buildings will present different scales, i.e., the same type of building may present different sizes on the same remote sensing image, which brings challenges to the fixed-size convolution kernel approach [22]. Image pyramids [21] and atrous convolution [23] provide a solution to this problem by generating multiscale image pyramids by downsampling images to different scales, enabling the model to learn features at different scales. Atrous convolution introduces a dilation rate on the basis of ordinary convolution, which increases the sensory field to different degrees by setting different dilation rates to capture contextual information at different scales without increasing the computational complexity. Finally, by fusing the features of different scales, the accuracy of the model in recognizing buildings of different scales is improved. In complex background environments, the influence of interfering objects such as roads, vegetation, and shadows, as well as the image blurring problem, results in blurred edges of buildings and increased pixel-value similarity with the surrounding background, making it more difficult to accurately distinguish buildings from the background. The self-attention mechanism in Transformer can effectively capture the global contextual information and calculate the correlation between each pixel and all other pixels in the image so as to effectively differentiate the overall information of the building and the background and improve the accuracy of the building extraction [24]. In addition, it is also possible to cope with the complexity of the background by increasing the interaction between the encoder and decoder and retaining high-resolution spatial information through skip connections [25].

In order to fully utilize the advantages of CNN and Transformer to achieve more accurate building extraction from remote sensing images, an asymmetric network named CTANet is proposed. It adopts CNN as the encoder and Transformer as the decoder and combines the advantages of the two in order to improve the accuracy and efficiency of building extraction. Specifically, in CTANet, the powerful ConvNeXt [26] is adopted as an encoder for feature extraction, which not only inherits the local feature extraction capability and parameter sharing property of traditional CNN, but also borrows many design principles from Transformer, such as an improved activation function and regularization method. This enables ConvNeXt to efficiently process multiscale features and adapt to changes in the scale of buildings in remote sensing images, thus enhancing its feature extraction capability. Next, an efficient bilateral hybrid attention transformer (BHAFormer) is designed as a decoder, which utilizes the advanced abstract feature processing capability of bilateral hybrid attention (BHA) to establish global dependencies from the perspectives of both texture edge features and background information, while reducing the length of the query, key, and value sequences to reduce the computational cost of the SA process. This design enables the model to cope better with interference from complex backgrounds and capture global information, thus improving the accuracy of edge extraction and overall structure recognition. Finally, information loss is a common problem during downsampling, especially during multiscale feature extraction, where detailed information is easily lost. To solve this problem, we introduce a multiscale mixed attention mechanism module (MSM-AMM). With MSM-AMM, the model is able to mitigate the information loss during downsampling and provide the decoder with multiscale semantic information and channel representations of the encoder features, thus preserving more detailed information and enhancing the accuracy of building extraction. The contributions of this paper can be summarized as follows:

(1) A Transformer block named bilateral hybrid attention transformer (BHAFormer) is proposed as a decoder. It establishes global dependencies from two perspectives: texture edge features and background information. At the same time, the BHAFormer reduces the computational cost of self-attention and improves the efficiency of building extraction.
(2) The multiscale mixed attention mechanism module (MSM-AMM) is designed to supplement the information loss in the downsampling process. This module contains two branches: the spatial attention branch (SAB), which utilizes strip convolution and 3 × 3 convolution with different depths to capture multiscale feature information, and the channel attention branch (CAB), which learns the correlation between channels through channel attention to suppress noise interference.
(3) Combining the above modules, an asymmetric network (CTANet) that combines CNN and Transformer is proposed. Compared to other state-of-the-art segmentation networks, CTANet achieves the best performance on three widely used building extraction datasets while keeping the computational cost low.

## 2. Related Work

This section explores recent relevant research results and model designs in remote sensing, focusing on analyzing the development of CNN-based semantic segmentation methods, Transformer in visual tasks, and its applications. These researches provide an important theoretical foundation and practical experience for our proposed approach.

**CNN-based semantic segmentation methods:** Convolutional neural networks (CNN) were first applied to image segmentation tasks owing to their powerful feature extraction capability, especially when dealing with local features such as image details and textures. In order to further improve the extraction of local features from images, a lot of research has been carried out. A fully convolutional network (FCN) [27] replaced the fully connected layer with a convolutional layer, which allowed the segmentation task to develop into an end-to-end pixel-level classification task, laying the foundation for FCN-based building extraction methods for remote sensing images. However, the spatial resolution of the FCN decreases during the downsampling process, resulting in the loss of detailed information and inability to fully capture detailed features such as building boundaries. U-Net [28] introduced an encoder-decoder structure. Image features are extracted in the encoder stage, the image spatial resolution is restored in the decoder stage, and encoder features are fused with decoder features through skip connections to better combine low-level spatial information with high-level semantic information. Deeplab [29] introduces atrous convolution and atrous spatial pyramid pooling (ASPP) techniques to enhance the capture of multiscale information by expanding the receptive field of the convolution kernel. DeeplabV3+ [30] further combines an encoder-decoder architecture to cope with more complex contexts. Similarly, PSPNet [21] employs a pyramid pooling module (PPM) to capture multiscale contextual information to improve semantic segmentation performance. PFPN [31] combines feature pyramid network (FPN) and panoramic segmentation concepts to enhance feature representation through multiscale feature fusion, which can cope with both object detection and semantic segmentation tasks.

CNNs are effective at extracting local features and learning rich semantic features. However, due to the fixed size of convolutional kernels in CNN-based models, their ability to capture long-range dependencies is limited. In addition, in remote sensing scenes with complex backgrounds, the complex shapes of buildings and occlusion of interfering objects may affect the capture of global features, which in turn affects the accuracy of building extraction.

**Transformer development in visual tasks:** Transformer was originally designed to solve natural language processing (NLP) problems. With its excellent global relational modeling capabilities, variants of Transformer quickly excelled in visual tasks and triggered a lot of research. The vision transformer (ViT) [32] was proposed to cope with the image task. The ViT segments an image into contiguous chunks, which are then spread out to serve as input to the Transformer. Through the self-attention (SA) mechanism, ViT can realize information exchange globally and capture long-distance dependencies in an image. Pyramid vision transformer (PVT) [33] introduces a pyramid structure that can handle the information at different scales. Meanwhile, PVT designed a spatial reduced attention (SRA) layer module to reduce the computational complexity by reducing the length of the key and value sequences in the SA computation. Swin Transformer [34] introduces the concept of shift windows, which divides the image into non-overlapping windows, computes SA locally within each window, and then obtains global attention by moving the window or stacking multiple Transformer blocks. Shunted Transformer [35] uses convolution kernels at different scales to convolve the input image and then obtains keys and values at different scales at different heads by multiscale token aggregation (MSTA) and linear transformation to capture the multiscale features of the image. However, these methods still maintain the full size of the query sequence when reducing the key and value sequences, thus still having high computational costs for SA computation. DSAFormer [36] significantly reduces the SA computational cost by simultaneously reducing the lengths of the query, key, and value sequences for coarse global relationship modeling. In addition, DSAFormer supplements the local spatial information by strip convolution. Although these Transformer variants perform well in image tasks, they still have some limitations: high computational cost and inferiority to traditional CNNs in capturing local detailed features.

**Application of Transformer in visual tasks:** Due to the high computational cost of Transformer, how to utilize Transformer effectively in semantic segmentation models and give full play to the advantages of both CNN and Transformer has become a hot research topic. Segmenter [37] and sparse token transformer (STT) [38] use Transformer in both the encoder and decoder parts to ensure that the global context information is fully captured at each stage. Swin-Unet [39] and DC-Swin [40] use the Swin Transformer in the encoder section to capture the details and global features of the image through a local window attention mechanism and multiscale feature fusion. TransUNet [41] uses a hybrid CNN-Transformer encoder, which first uses CNN for feature extraction, then the extracted feature maps are converted into sequences by patch embedding, which is used as input to the Transformer to capture global contextual information. TCNet [42], TransFuse [43], and BuildFormer [44] all use a hybrid architecture combining a CNN and Transformer to extract both local features and global contextual information using a dual backbone, followed by multiscale feature fusion to improve the model’s ability to recognize buildings of various sizes.

Despite the excellent results achieved by the above models, these models usually employ Transformer blocks in the encoder stage, or throughout the model, or use a dual-backbone structure of CNN and Transformer, which requires a large number of parameters and high computational cost. Considering the respective advantages of CNN and Transformer, we propose an asymmetric network called CTANet that achieves the best extraction performance on three building datasets at a low computational cost compared to other state-of-the-art segmentation networks. In addition, we have briefly summarized the strengths and weaknesses of the aforementioned methods in Table 1, which provides valuable insights for the design of our proposed approach.

## 3. Methods

This section first describes the general architecture of the proposed asymmetric network CTANet and then describes in detail the modular structure we used in each part of the model separately, including the ConvNeXt block used in the encoder stage, the BHAFormer block used in the decoder stage, and the MSM-AMM introduced in the middle of the model. Finally, a loss function is introduced.

### 3.1. General Architecture of CTANet

**Figure 2:**
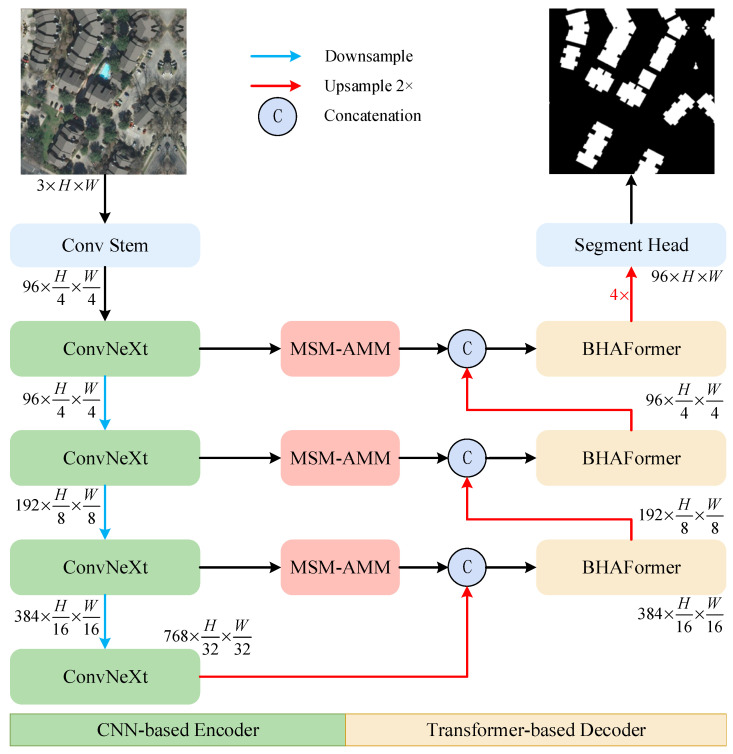
General structure of CTANet.

As shown in Figure 2, our proposed CTANet is an asymmetric network designed following the encoder-decoder structure. The network mainly consists of ConvNeXt, BHAFormer, and MSM-AMM.

In the encoder stage, we use the powerful CNN model ConvNeXt-Tiny as the backbone network and utilize the local feature extraction capability and parameter sharing property of CNN to first extract features from the image. As the depth of the convolutional layers increases, different convolutional layers can extract the features at different semantic levels. Among them, shallow features contain rich fine-grained information and are mainly used to capture details and local features, which can accurately capture detailed information such as the colors, textures, edges, and corners of buildings. Deep features, on the other hand, contain more high-level abstract information, mainly coarse-grained information, i.e., semantic information, which is used to extract high-level abstract features, such as the global shape and structure of the building, as well as the category and spatial relationship of the building. The combination of shallow and deep features will help improve the accuracy and completeness of building extraction.

In the decoder stage, we use BHAFormer for post-processing to compute global information. The deep feature output from the encoder is used as the input to the BHAFormer in the decoder, and the global information is modeled using the high-level abstract feature processing capabilities of the two attention paths of the BHAFormer to capture more complex semantic features. BHAFormer contains significant attention path (SAP) and overall attention path (OAP), in which the size of the input feature map can be reduced to 1/4 of its original size and retained to contain significant features such as texture, edges, and corners, as well as overall features such as overall structure and background information, respectively, before SA computation. In this way, it is possible to realize global relationship modeling from different perspectives separately, while reducing the cost of SA computation by exploring global dependencies from more perspectives, which helps to extract the boundary of a building more accurately and locate the building as a whole in a variety of complex scenarios. In addition, by introducing MSM-AMM between the encoder and the decoder, the information loss caused by the downsampling process is mitigated, and the decoder is provided with the multiscale semantic information and channel representation of the encoder.

The MSM-AMM processed output is spliced with the output of the previous layer of the corresponding layer of the decoder to fuse the shallow features with the deeper ones through two 3 × 3 convolutions, which helps improve the accuracy and completeness of the building extraction. After that, it is used as the input of the corresponding layer BHAFormer for global relationship modeling, and finally, the feature map is restored to its original size by the upsampling module to obtain the final building extraction result. The module optimizes the features, suppresses noise interference, and enhances building-related features. To more clearly illustrate the changes in image size within each layer of the network, we have created an architecture table that sequentially numbers each layer based on the flow of the image. The table includes the layer name, input and output sizes, and filters, as shown in Table 2.

### 3.2. ConvNeXt

Although CNN networks perform well in tasks such as image classification and target detection, variants of Transformer even outperform traditional CNNs in visual tasks due to Transformer’s superior performance in capturing global contextual information and modeling complex dependencies. ConvNeXt is proposed to address the limitations of traditional CNNs in complex visual tasks. It borrows some design concepts from the Swin transformer and optimizes the CNN architecture, which allows ConvNeXt to retain the powerful feature extraction capability and computational efficiency of the CNN while increasing the receptive field and improving the global information capture capability of the model, which is more suitable for the complex visual tasks nowadays.

**Figure 3:**
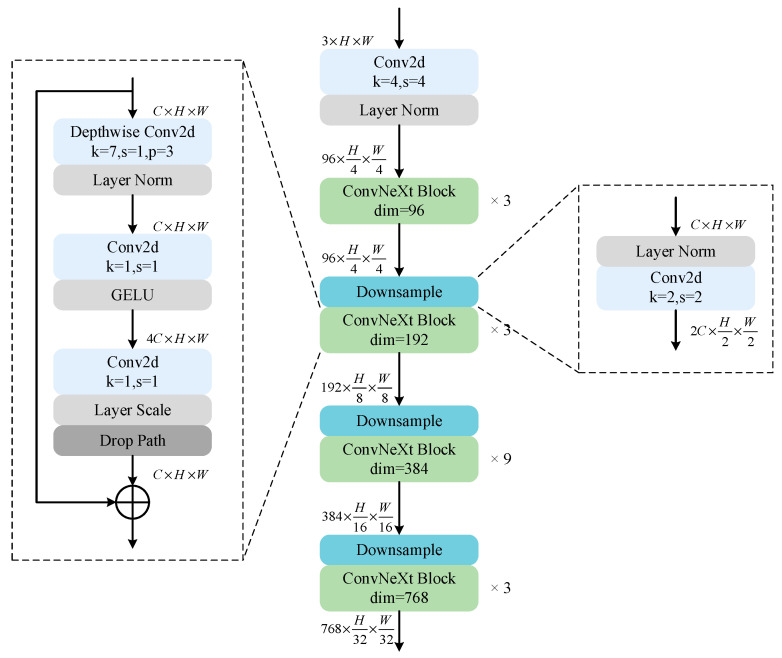
Detailed structure of ConvNeXt.

As shown in Figure 3, the ConvNeXt backbone network has a hierarchical structure divided into four stages, each consisting of multiple convolutional blocks (ConvNeXt blocks). Among them, the number of ConvNeXt block stacks in each stage is 3, 3, 9, and 3. At the entrance of the network, the image is first passed through a convolutional layer with a step size of 4 to fuse neighboring elements and reduce the image size. After that, before the beginning of each stage, downsampling is performed through a convolutional layer with a step size of 2 to extract features at different scales. With the design of a layered structure, ConvNeXt is able to better capture shallow features, such as the details and edges of buildings, as well as deeper features, such as the overall structure and layout.

The ConvNeXt block is similar in design to the inverted bottleneck structure of MobileNetV2 [45], but the location of the convolutional layers is different. Feature extraction is first performed by depthwise convolution with the number of groups equal to the number of input channels, and each convolutional kernel is responsible for only one channel, reducing the number of parameters and achieving a balance between FLOPs and accuracy. Unlike traditional convolution, which uses a 3 × 3 convolution kernel, ConvNeXt uses a larger 7 × 7 deep convolution kernel, which enhances the model’s receptive fieldand improves its ability to capture global information. The number of channels of the feature map is then expanded by a 1 × 1 convolution, and another 1 × 1 convolution is used to compress the number of channels of the feature map back to its original dimension, increasing the feature representation capability of the network. In addition, ConvNeXt has undergone several adjustments to further optimize performance. For example, the number of activation and normalization layers is reduced, and layer normalization (LN) is used instead of batch normalization (BN), which helps reduce the computational overhead and memory usage during training. At the same time, the GELU activation function is used instead of the ReLU activation function, which enhances the nonlinear expression ability of the model and improves training stability and overall performance. Through this fine design and optimization, ConvNeXt is able to achieve higher feature extraction and information processing capabilities while maintaining computational efficiency.

### 3.3. BHAFormer

The SA process in the conventional vision transformer (ViT) flattens the input 2D image ($X\;\in R^{C\times H\times W}$) into a sequence of 1D image blocks ($X\in R^{N\times C}$) and performs a linear mapping using three learnable weight matrices ($W_{q}$, $W_{k}$, and $W_{v}\in R^{C\times d_{k}}$) to obtain the query (*Q*), key (*K*), and value (*V*) sequence vectors from the input image sequences, all of which have dimensions *N* × $d_{k}$. Next, the attention score matrix, with dimensions *N* × *N*, is generated by performing a dot product operation on the tokens of the query and key sequences. This matrix reflects the similarity information between different image blocks (tokens). To prevent the result from being too large, it is divided by a scaling factor $\sqrt{d_{k}}$, where $d_{k}$ denotes the dimension of the vector, and *N* = *H* × *W* denotes the length of the sequence of image blocks. Then, the attention scores are converted into an attention weight matrix using the softmax function, which represents the relative importance of individual image blocks. Finally, the attention weight matrix is multiplied by a sequence of values to perform a weighted summation so that the model can focus on the most relevant information to establish global dependencies. The self-attention (SA) mechanism ensures the effective interaction of global information in the input image sequence by performing the above computation for each token. The SA computation can be expressed as the following equations:

(1) $$Q,K,V={XW}_{q},{XW}_{k},{XW}_{v}$$

(2) $$\mathrm{Attention}(Q,K,V)=\mathrm{softmax}(\frac{{QK}^{T}}{\sqrt{d_{k}}})V$$

However, since the SA computational complexity is quadratic with respect to the sequence length, this brings about a huge amount of computation. In order to reduce the computational cost of processing high-resolution images, some visual transformers have attempted to optimize the SA computation process. For example, PVT designed spatial-reduction attention (SRA) to reduce the sequence length of keys and values; Shunted Transformer designed shunted SA (SSA) to downsample keys and values with different heads to different sizes in order to obtain features with different granularities. Although these methods reduce the sequence length of keys and values, simple downsampling leads to a loss of information, which affects the global relationship modeling of important features, such as building texture, edges, and corner points. In addition, the query in these methods remains full size, causing the feature maps obtained from SA computation to remain the same size as the query, keeping the computational cost high.

**Figure 4:**
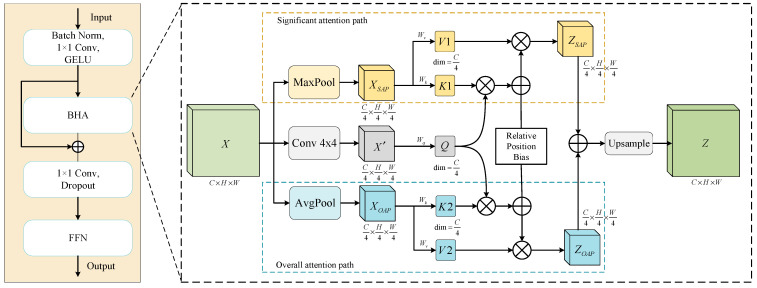
The structure of BHAFormer block. The right side of the figure shows the details of the bilateral hybrid attention (BHA).

Previous work pointed out [36] that intensive pixel-level SA computation may not be required for the global relationship modeling of individual buildings in a local area. Therefore, it performs rough global relationship modeling by reducing the query, key, and value sequence lengths; however, this cannot be used to treat local details. To address these problems, we designed the BHAFormer. As shown in Figure 4, the BHAFormer block consists of a batch normalization layer, two 1 × 1 convolutional layers, a GELU activation layer, a dropout layer, bilateral hybrid attention (BHA), and a feed-forward network layer.

Considering the positive significance of pooling operations in feature extraction, maximum pooling can highlight the significant features in the image, such as texture, edges, and corner points, while average pooling can preserve the overall data features and highlight the overall structure and background information of the image. Therefore, the BHA designed two paths of attention. First, we subject the input image $X\in R^{C\times H\times W}$ to a spatial-reduction operation (SR) by fusing neighboring pixels through a 4 × 4 convolution to obtain a feature map $X\prime \in R^{\frac{C\times H\times W}{4}}$ with a size of 1/4 of the original image. Then, the feature map is flattened into a sequence of *N* × *C*/4, and the query ($Q\in R^{N\times d_{k}}$) sequence vector is obtained using the weight matrix $W_{q}\in R^{\frac{C}{4}\times d_{k}}$ mapping to shorten the sequence length, where *N* = *H*/4 × *W*/4. Next, the feature map *X* is subjected to a maximum pooling operation via the significant attention path (SAP) to highlight texture, edge, and corner features to obtain $X_{SAP}\in R^{\frac{C\times H\times W}{4}}$. Meanwhile, an average pooling operation is performed through the overall attention path (OAP) to highlight the background information and the overall structure to obtain $X_{OAP}\in R^{\frac{C\times H\times W}{4}}$. Here, the window sizes are both set to 4. Subsequently, $X_{SAP}$ and $X_{OAP}$ are linearly transformed through the weight matrices $W_{k}$ and $W_{v}\;\in R^{\frac{C}{4}\times d_{k}}$, respectively, to obtain the sequence vectors *K*1, *V*1, and *K*2, $V2\in R^{N\times d_{k}}$. These two sets of keys and values are then used in SA computation with the query (*Q*) to establish global dependencies from the perspectives of texture edge features and overall background information, respectively. To better capture the relative position information, the relative position encoding ($B\in R^{N\times N}$) is added after the dot product of *Q* with *K*1 and *K*2, and then multiplied with *V*1 and *V*2, respectively, to obtain the weighted feature maps $Z_{SAP}$ and $Z_{OAP}\in R^{\frac{C\times H\times W}{4}}$. Finally, the fused $Z_{SAP}$ and $Z_{OAP}$ are upsampled to their original dimensions to obtain the final output $Z\in R^{C\times H\times W}$, which is then conveyed to the feed-forward network. The process of BHA can be represented by the following equations:

(3) $$X^{\prime }\;=\;\mathrm{SR}(X)$$

(4) $$X_{SAP},X_{OAP}=\mathrm{MaxPool}(X),\mathrm{AVgPool}(X)$$

(5) $$Q={X^{\prime }\;W}_{q}$$

(6) $$K1,V1=X_{SAP}W_{k},X_{SAP}W_{v}$$

(7) $$K2,V2=X_{OAP}W_{k},X_{OAP}W_{v}$$

(8) $$\mathrm{Attention}(Q,K,V)=\mathrm{softmax}(\frac{{QK}^{T}}{\sqrt{d_{k}}}+B)V$$

(9) $$Z_{SAP},Z_{OAP}=\mathrm{Attention}(Q,K1,V1),\mathrm{Attention}(Q,K2,V2)$$

(10) $$Z\;=\;\mathrm{Upsample}(Z_{SAP}+Z_{OAP})$$

### 3.4. MSM-AMM

**Figure 5:**
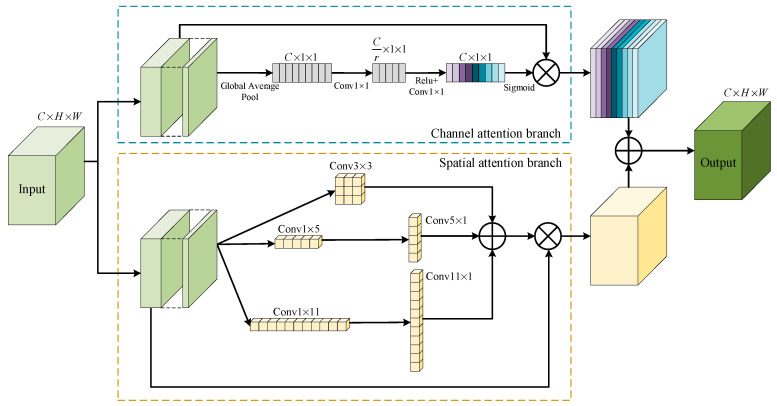
The structure of MSM-AMM. Contains spatial attention branch and channel attention branch.

During the downsampling process of the encoder, the shallow features generated contain a wealth of fine-grained information, such as color, texture, edges, and corner points, which are important. However, as the network layers deepen, it inevitably leads to information loss and noise interference. To solve this problem, we design the multiscale mixed attention mechanism module (MSM-AMM), which is used to supplement the information loss in the downsampling process and enhance the network’s ability to recognize buildings. As shown in Figure 5, MSM-AMM contains two attention branches for enhancing the multiscale representation of encoder features in the spatial semantic and channel dimensions. In the spatial attention branch, we use a 3 × 3 convolution and two sets of strip convolutions (1 × 5 and 5 × 1, 1 × 11, and 11 × 1) to capture multiscale features. Strip convolutions are particularly suitable for extracting strips from segmented scenes [46]. In the channel attention branch, the feature map is compressed to 1 in length and width by global average pooling to efficiently aggregate the global spatial information of each channel so as to pay more attention to the representation of channel features. Next, we use two 1 × 1 convolutions to compress the number of channels to 1/r times the original and then recover them to learn the dependencies between channels, highlight important features, suppress unimportant features, and reduce the noise, where the compression ratio r is 16. Then, the channel attention map is obtained by a sigmoid function, which is used to further adjust the importance weights of each channel. Finally, the outputs of the two branches are multiplied by the original inputs to learn a multiscale representation of the space and channel dimensions, which are then added together to fuse the features.

### 3.5. Loss Function

During the model training process, we adopted the same approach as in [44], using a joint loss function for optimization, which includes a cross-entropy loss and a dice loss, to guide the model to correctly classify each pixel in the image as either a building or a background. In addition, we introduced binary cross entropy as an edge loss to ensure that the model focuses on the accurate extraction of the building edge pixels during the training process and reduces edge blurring, thus improving the accuracy of the building boundary extraction. The specific loss function formula can be expressed as follows:

(11) $$L_{total\;}=\alpha L_{CE}+\beta L_{Dice}+\gamma L_{BCE}$$

where $L_{CE}$ denotes cross-entropy loss, $L_{Dice}$ denotes dice loss and $L_{BCE}$ denotes binary cross-entropy loss. In our experiments, we set the parameters α, β, and γ to 1.0, 1.0, and 10.0, respectively.

## 4. Experiments

In this section, we first introduce the three widely used building datasets [9,10,11] on which we conduct our experiments in this paper and the related experimental setup we employ. Then, ablation studies are performed on our proposed BHAFormer and MSM-AMM to demonstrate their effectiveness. Finally, we select the most recent state-of-the-art semantic segmentation models for comparison experiments with our proposed model to demonstrate the superiority of our proposed CTANet.

### 4.1. Dataset Introduction

#### 4.1.1. Inria

The Inria aerial image labeling dataset contains scenes from ten cities, each with 36 RGB images of 5000 × 5000 pixels with a ground sampling distance of 0.3 m. The training set contains five cities, Austin, Chicago, Kitsap, Tyrol, and Vienna, and the test set contains five cities: Bellingham, Bloomington, Innsbruck, San Francisco, and East Tyrol. We refer to [44] as well as the official partitioning recommendations and select the first five images of each city in the original training set for validation and the rest for model training. Before training, we fill each image with 5120 × 5120 pixels and then crop it into 512 × 512 pixel blocks for training.

#### 4.1.2. Massachusetts

The Massachusetts building dataset consists of 151 urban aerial images measuring 1500 × 1500 pixels in the Boston area of the United States and contains a wide range of scales, shapes, and types of building facilities in the area. The entire dataset covers about 340 km^2^ of the Boston area, with a ground sampling distance of 1 m. Before model training, we fill each image and then crop it into 512 × 512 pixel image blocks for the experiments.

#### 4.1.3. WHU

The WHU building dataset covers the city of Wuhan, China, and contains both high-resolution satellite imagery and aerial imagery. The aerial image dataset covers an area of more than 450 km^2^ and involves 22,000 buildings. The image size is 512 × 512 pixels, and the ground sampling distance is 0.3 m. In this paper, we select the aerial image dataset for the experiments.

### 4.2. Experimental Details

#### 4.2.1. Experimental Settings

In this paper, our experiments are conducted on a single NVIDIA GTX 3090 GPU (24G RAM; Nvidia, Santa Clara, CA, USA) using the PyTorch 1.10.0 framework. Experimentally, we use the AdamW optimizer with an initial learning rate set to $1\times 10^{-3}$ and a weight decay of 0.0025. The learning rate scheduling strategy is used to train the model using a cosine annealing strategy for a total of 105 training epochs. In order to expand the dataset, we augmented the data with random flip, random brightness and contrast change, and random rotation for all training samples to enhance the generalization ability of the model. For the test samples, we applied horizontal and vertical flipping. These settings partly refer to the experimental design in [44].

#### 4.2.2. Evaluation Metrics

We evaluate the performance of the model using four metrics that are widely used in the field of building extraction, i.e., *Precision*, intersection over union (*IoU*), *Recall*, and *F*1-*score*. *Precision* indicates which of the predicted pixels are real building pixels. *IoU* indicates the overlap between predicted building pixels and real building pixels. *Recall* indicates which of the real building pixels are correctly predicted. The *F*1-*score* combines precision and recall to provide an overall measure. A higher *F*1-*score* indicates better performance in terms of both precision and recall. Each metric can be expressed by the following equation:

(12) $$Precision=\frac{TP}{TP+FP}$$

(13) $$IoU=\frac{TP}{TP+FP+FN}$$

(14) $$Recall=\frac{TP}{TP+FN}$$

(15) $$F1-score=2\times \frac{Precision\times Recall}{Precision+Recall}$$

where true positive (*TP*) indicates the number of pixels correctly predicted as buildings, i.e., the model correctly identifies the area of the building. A false positive (*FP*) denotes the number of pixels incorrectly predicted as buildings, i.e., the pixel label is not a building, and the model incorrectly predicts it as a building pixel. A false negative (*FN*) indicates the number of pixels not predicted to be buildings, i.e., the pixels of actual buildings are incorrectly predicted by the model as background and other distractions.

### 4.3. Ablation Study

In order to fully evaluate the effectiveness of each module in our proposed CTANet, we chose to perform ablation experiments on the Inria aerial image labeling dataset and the Massachusetts building dataset, as shown in Table 3 and Table 4. Compared to the Massachusetts dataset, the Inria dataset is more representative of model training and evaluation due to its diversity and high resolution. At the same time, a larger image size contains more types of urban buildings, which makes the experimental results more generalizable. Therefore, we analyze the experimental results of the Inria dataset in detail.

**Effectiveness of BHAFormer:** As shown in Table 3, the experiment was first tested using the benchmark model ConvNeXt-Tiny, which yielded an F1-score, IoU, and precision of 89.81%, 81.51%, and 91.17%, respectively. When we add BHAFormer as a decoder to the benchmark model, the results show that the F1-score, IoU, and precision are 90.37%, 82.43%, and 91.58%, respectively, which are improved by 0.56%, 0.92%, and 0.41% over the benchmark model. This indicates that BHAFormer is more accurate in extracting the edges and complex structures of buildings by establishing global dependencies from both texture edge features and overall background information. This improves the classification performance of the model as a whole, thus proving the effectiveness of the module.

**Effectiveness of MSM-AMM:** Similarly, when we add only MSM-AMM to the baseline model, the results show that the F1-score, IoU, and precision are 90.26%, 82.25%, and 91.43%, respectively. Compared to the baseline model, there is an improvement of 0.45%, 0.74%, and 0.26%, respectively, thus proving the effectiveness of MSM-AMM.MSM-AMM improves the accuracy of the model in recognizing buildings of different sizes and shapes by capturing multiscale features, thus improving the overall segmentation performance.

**Figure 6:**
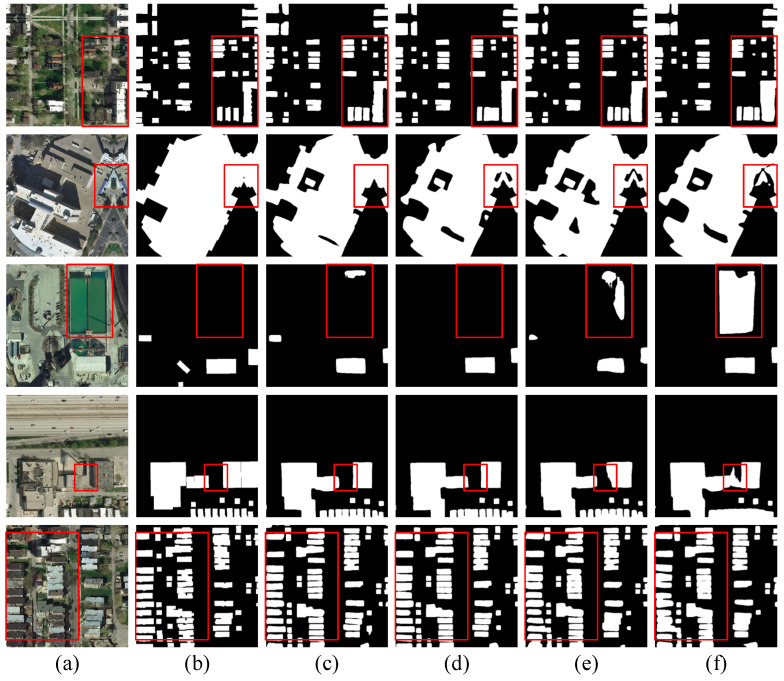
Visualizing ablation study results on the Inria aerial image labeling dataset. (**a**) Images. (**b**) Labels. (**c**) CTANet. (**d**) Only BHAFormer. (**e**) Only MSM-AMM. (**f**) Baseline.

Subsequently, we added BHAFormer and MSM-AMM to the benchmark model, and the results showed that the F1-score, IoU, and precision were 90.52%, 82.68%, and 91.75%, respectively, for the best performance. Compared to the benchmark model, the three evaluation metrics improved by 0.71%, 1.17%, and 0.58%, respectively. These results show that the comprehensive performance of the model is significantly improved by combining BHAFormer and MSM-AMM, which further proves that each of our proposed modules helps the model in building extraction. As shown in the visualization results in Figure 6, before the introduction of BHAFormer and MSM-AMM, the segmentation results of the baseline model showed more mispredictions by predicting the background as buildings, while the edges of the closely spaced small buildings were lost, resulting in the display of a blurred whole in the prediction result map. When BHAFormer and MSM-AMM are added, the mispredictions of buildings are reduced, edges are smoother, and the gaps between buildings in a dense cluster of small buildings are more clearly visible. This illustrates that the global relationship modeling capability of BHAFormer helps the model to accurately locate buildings in complex background environments and reduces false predictions. Meanwhile, the multiscale features captured by MSM-AMM enable the model to extract small buildings more accurately and suppress the interference of background noise on building edge extraction.

### 4.4. Experimental Results and Analysis

To demonstrate the superiority of our proposed CTANet, we conduct comparative experiments with recent state-of-the-art methods on three widely used datasets ( the Inria aerial image labeling dataset, Massachusetts building dataset, and WHU building dataset). The selected methods include semantic segmentation models: Segmenter [37], Swin-Unet [39], DC-Swin [40], TransUNet [41], and TransFuse [43], as well as models specialized for building extraction: MSRF-Net [6], STT [38], TCNet [42], and BuildFormer [44].

#### 4.4.1. Experiment on the Inria Aerial Image Labeling Dataset

As shown in Table 5, on the Inria aerial image labeling dataset, our proposed CTANet achieved the best results on four evaluation metrics, with an F1-score, IoU, precision, and recall of 90.52%, 82.68%, 91.75%, and 89.32%, respectively, which were significantly higher than those of other state-of-the-art methods. Specifically, compared to the best-performing semantic segmentation models TransUNet and Swin-Unet, CTANet is 1.16% and 3.98% higher in F1-score, 1.91% and 6.41% higher in IoU, 1.31% and 4.88% higher in precision, and 1.0% and 3.11% higher in recall, respectively. Meanwhile, compared with BuildFormer, a state-of-the-art method focusing on building extraction, our F1-score, IoU, precision, and recall are 0.75%, 1.24%, 1.0%, and 0.51% higher, respectively. These results demonstrate the superior performance of CTANet in building extraction. Although the FLOPs of CTANet are slightly higher than those of TransFuse, it brings significant improvement in the four evaluation metrics and has significantly lower FLOPs than the other methods.

**Figure 7:**
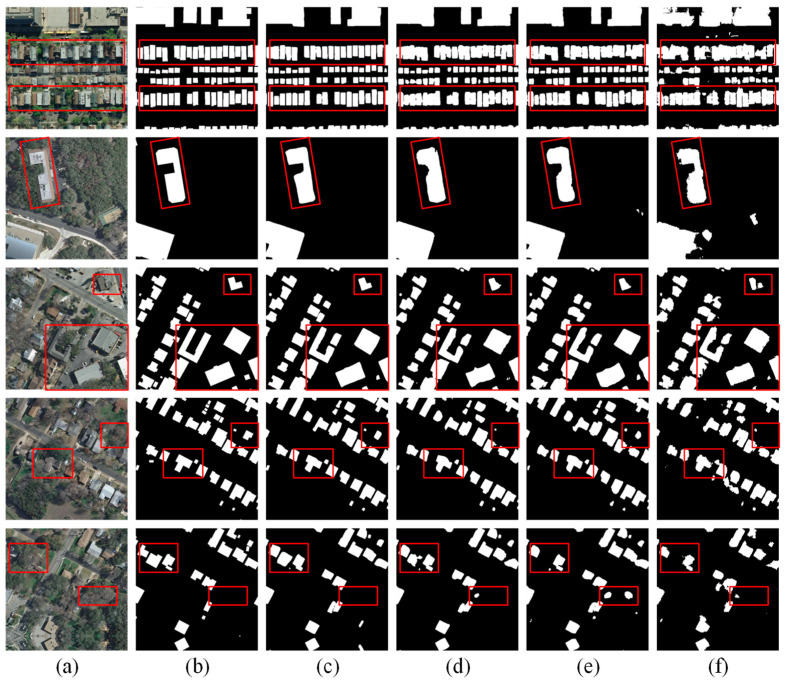
Visualization results of CATNet and state-of-the-art methods on the Inria aerial image labeling dataset. (**a**) Images. (**b**) Labels. (**c**) CTANet. (**d**) BuildFormer. (**e**) TransUNet. (**f**) Swin-Unet.

As shown in Figure 7, we visualize and compare the prediction results of CTANet with those of the recent state-of-the-art Transformer-based methods (TransUNet, Swin-Unet, and BuildFormer). It can be seen that in the second row, although the buildings are less disturbed by the background, TransUNet, Swin-Unet, and BuildFormer perform poorly compared to CTANet in terms of the building edge extraction results, and even make wrong predictions, mistaking the background for the buildings. This is because CTANet uses ConvNeXt as an encoder, which is good at detail and local feature extraction, while the large convolutional kernel ensures a certain global information-capturing ability, which is especially important for the recognition of building edges and complex structures. As can be seen from the results in the first and third rows, when facing a dense cluster of small buildings, CTANet is able to extract the edges of the buildings more clearly and smoothly with the help of MSM-AMM, reflecting the independence between the buildings. While other models usually cannot effectively recognize the gaps and edge pixels between buildings when facing dense building clusters, resulting in redundant and irregular extraction results. In the results of the fourth and fifth rows, it can be seen that there is multiple vegetation in the background to cover the buildings, which leads to the omission of other models in extracting the buildings, incorrectly recognizing the buildings as the background, and failing to represent the overall structure of the buildings in a complete way. In contrast, CTANet, with the help of BHAFormer, is able to effectively locate the buildings in a complex background environment and accurately distinguish the overall differences between the buildings and the background.

#### 4.4.2. Experiment on the Massachusetts Building Dataset

As shown in Table 6, CTANet outperforms other state-of-the-art methods on the Massachusetts building dataset, achieving the highest F1-score, IoU, and precision of 86.7%, 76.52%, and 88.2%, respectively. Specifically, CTANet achieves 0.35% and 0.78% higher F1-score, 0.54% and 1.21% higher IoU, and 1.8% and 0.5% higher precision, respectively, compared to the best-performing semantic segmentation models, Swin-Unet and TransUNet. Although lower than Swin-Unet and the latest building extraction network TCNet in the recall, the F1-score combines precision and recall and is more representative in measuring the comprehensive performance of the model. Compared to BuildFormer, the best-performing building extraction network, CTANet’s F1-score is 0.51% higher, IoU is 0.78% higher, precision is 0.68% higher, and recall is 0.35% higher. In addition, CTANet maintains the lowest FLOPs, showing the advantage of its computational efficiency.

**Figure 8:**
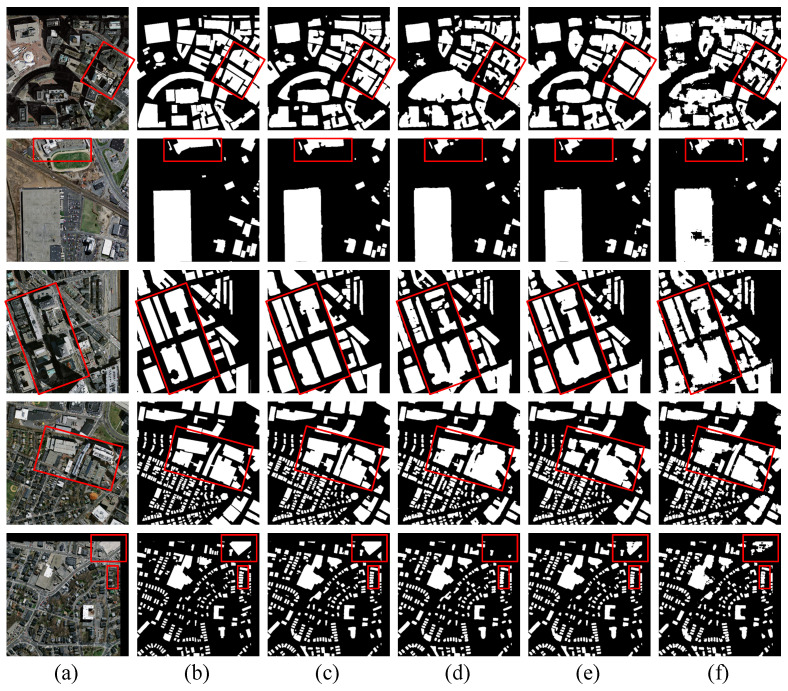
Visualization results of CATNet and state-of-the-art methods on the Massachusetts building dataset. (**a**) Images. (**b**) Labels. (**c**) CTANet. (**d**) BuildFormer. (**e**) TransUNet. (**f**) Swin-Unet.

Figure 8 shows the visualization results of CTANet with other models on the Massachusetts building dataset. The buildings in the Massachusetts dataset have uneven density and distribution and are affected by lighting conditions, which leads to variations in the brightness and contrast of the images, making the extraction of buildings more difficult. As can be seen from the images in the first and third rows, the buildings have complex structures and are affected by lighting, and the shadow coverage makes the buildings similar to the background in terms of grayscale values, losing the texture and color features of the buildings. These factors cause other models to incorrectly identify shadowed areas as buildings or fail to recognize the parts of buildings that are covered by shadows. CTANet combines global and local information to enhance the robustness of the model to the shadow-covered areas, preserving as much as possible the original structure of the buildings. In the images of the second, fourth, and fifth rows, due to the blurring of the dataset images, when the color and texture of the buildings are similar to the background, noise interference is introduced, which may result in missed detection or incorrect prediction, and increase the difficulty of building extraction. CTANet enhances the features and reduces the noise to preserve the integrity of buildings through multiscale learning of the spatial and channel dimensions.

#### 4.4.3. Experiment on the WHU Building Dataset

To demonstrate the generalization ability of CTANet in different environments, we continue to compare CTANet with other state-of-the-art methods in our experiments on the WHU building dataset. As shown in Table 7, CTANet still achieves the best evaluation results of 95.74%, 91.84%, 95.86%, and 95.63% for the F1-score, IoU, precision, and recall, respectively. Compared to Swin-Unet and TransUNet, CTANet has 1.28% and 2.17% higher F1-score, 2.34% and 3.93% higher IoU, 1.33% and 2.41% higher precision, and 1.25% and 1.94% higher recall. Compared to the best-performing building extraction network, BuildFormer and CTANet have 0.21%, 0.4%, 0.21%, and 0.23% higher F1-score, IoU, precision, and recall, respectively. These results fully demonstrate that CTANet possesses strong generalization ability and balanced performance.

**Figure 9:**
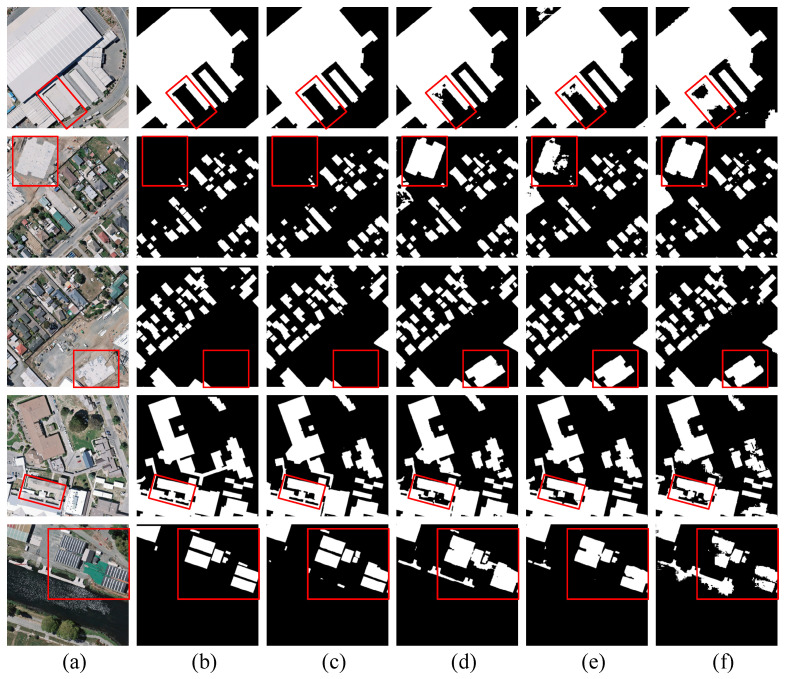
Visualization results of CATNet and state-of-the-art methods on the WHU building dataset. (**a**) Images. (**b**) Labels. (**c**) CTANet. (**d**) BuildFormer. (**e**) TransUNet. (**f**) Swin-Unet.

Figure 9 shows the prediction results of CTANet compared with those of the other models on the WHU building dataset. As can be seen from the images in the first, second, and third rows, the similarity in color and the lack of texture between the parcels and the buildings cause most of the models to incorrectly predict the parcels as buildings. This is because color is a very important feature in visual tasks and dominates the process of learning and building features using a model. If this feature is overly relied upon and other features are ignored, there will be cases in which backgrounds with similar colors are incorrectly identified as buildings. CTANet enhances the model’s ability to extract diverse features of a building through multiscale feature extraction and attention mechanisms to improve the recognition accuracy. As can be seen from the images in the fourth and fifth rows, the traditional square convolutional kernel may not be effective in performing feature extraction for more slender and narrow spaghetti buildings. At the same time, the small number of samples for this type of building makes it impossible for models with poor generalization ability to effectively identify and segment these buildings. CTANet enhances the ability to capture slender strip features through strip convolution with different depths and multiscale feature fusion, providing enough details to extract the buildings accurately.

## 5. Discussion

To further validate the superiority and necessity of our proposed module, we replace the BHAFormer in the decoder part with other excellent Transformer modules, such as PVT [33], Shunted Transformer [35], and DSAFormer [36], while keeping the CTANet network architecture unchanged, and conducted experiments on the Inria aerial image labeling dataset. As shown in Table 8, when CTANet introduces BHAFormer in the decoder, the F1-score and IoU achieve the best performance while maintaining the lowest FLOPs and number of parameters, which fully proves the high efficiency and lightweight of BHAFormer. Although PVT and Shunted Transformer reduce the cost of SA computation by reducing the length of the key and value sequences, the length of their query sequences remains full size, which keeps the computational cost high during SA computation. DSAFormer reduces the length of the query, key, and value sequences in SA computation by downsampling the image; however, the ordinary downsampling operation leads to loss of information and thus needs to be supplemented by additional local feature extraction. Owing to the bilateral hybrid attention mechanism, BHAFormer reduces the length of the query, key, and value sequences to reduce the cost of SA computation while preserving the local features, such as edges, textures, and corner points, as well as global features, such as the overall structure and background information, and performs global dependency modeling from two perspectives, which achieves the best performance of building extraction.

Next, we continue to replace MSM-AMM with a skip connection while keeping the overall architecture of CTANet unchanged. The experimental results are shown in Table 9, where the performance is improved after replacing the skip connection; however, the results are still lower than those of CTANet with MSM-AMM. This is because loss of information is unavoidable during the encoder downsampling process. The skip connection connects the encoder to the decoder to enhance information interaction, supplement the decoder with shallow features, and improve segmentation performance, which demonstrates the positive significance of fusing shallow features with deep features for building extraction. However, unprocessed encoder features contain noise interference and lack multiscale information. MSM-AMM further improves the segmentation accuracy by capturing multiscale feature information through a spatial attention branch and channel attention branch, learning channel representations, and reducing noise.

## 6. Conclusions

In this paper, we propose an asymmetric network that combines the advantages of CNN and Transformer, named CTANet, to realize the accurate extraction of buildings. CTANet uses ConvNeXt as the encoder, and BHAFormer as the decoder. Compared to general Transformers, BHAFormer improves efficiency by reducing the computational cost of self-attention while establishing global dependencies from two perspectives: texture and edge details, as well as overall background information. At the same time, we introduce MSM-AMM to capture multiscale feature information and learning channel representations to reduce noise and complement information loss during downsampling to improve the segmentation performance of buildings. These innovations make CTANet stand out in comparison with state-of-the-art methods, performing well on three widely used datasets: the Massachusetts building dataset, the WHU building dataset, and the Inria aerial image labeling dataset. CTANet not only maintains a relatively low computational cost, but also excels in segmenting building edges, especially in dense building clusters.

It is worth noting that the method proposed in this paper still has some limitations; for example, complex dynamic environments, such as light changes, shadow coverage, and seasonal changes, still have an important impact on the extraction effect of buildings. In the future, we will further delve into the network structure such that the model can effectively capture both local and global features at each stage and improve the generalization ability of the model in changing environments. In addition, we will continue to keep the computational cost low to meet the needs of practical applications.

## Figures and Tables

**Table 1:** Brief summary of related work.

Method	Advantages	Disadvantages
FCN [27]	End-to-end pixel-level classification.	Loss of fine details due to resolution reduction during downsampling.
U-Net [28]	Encoder-decoder structure with skip connections enhances feature fusion between low-level spatial and high-level semantic information.	Limited performance in complex scenes and when the dataset is small or not diverse enough.
Deeplab series [29,30]	Uses atrous convolution and ASPP to enhance multiscale feature extraction and captures complex contexts effectively.	Increased computational complexity and slower inference speed.
PSPNet [21]	Pyramid pooling module (PPM) improves multiscale contextual information capture.	Still suffers from accuracy loss on the edges of very small objects.
PFPN [31]	Combines feature pyramid network (FPN) with panoptic segmentation to enhance multiscale feature representation.	High computational resource requirements, slow training, and inference.
ViT [32]	Excels in capturing global dependencies via self-attention.	High computational cost; struggles with local feature extraction.
PVT [33]	Pyramid structure handles multiscale information; SRA mechanism reduces computational complexity.	Less effective than CNNs in local feature extraction.
Swin Transformer [34]	Uses a shifting window mechanism, effectively capturing both local and global information.	Long training time for large-scale images, high memory consumption.
Shunted Transformer [35]	Improves model efficiency and accuracy through multiscale token aggregation.	Limited performance on small datasets has a large number of parameters.
DSAFormer [36]	Significantly reduces self-attention computation by shortening query, key, and value sequences.	Less capable of capturing fine local features compared to CNN-based methods.
Segmenter [37]	Uses Transformer in both encoder and decoder to capture global context at every stage.	Large number of parameters and high computational demand.
STT [38]	Uses sparse token processing to reduce computational load and improve efficiency.	Weak in capturing local features.
Swin-Unet [39]	Employs Swin Transformer to capture both local and global information.	High computational overhead and long training time.
DC-Swin [40]	Combines Swin Transformer for fine-resolution remote sensing image segmentation.	Requires significant computational resources for high-resolution images.
TransUNet [41]	Hybrid CNN-Transformer encoder captures both local and global information.	Complex architecture, resulting in high inference time and resource consumption.
TCNet [42]	Dual-backbone architecture enhances the recognition of buildings of various sizes.	Large number of parameters; high computational burden.
TransFuse [43]	Hybrid CNN and Transformer architecture capture both local features and global contextual information.	High computation due to dual-backbone design.
BuildFormer [44]	Dual-backbone architecture effectively combines local and global feature extraction.	High computational cost due to the complexity of Transformer components.
Proposed CTANet	Combines the strengths of CNN and Transformer in a hybrid architecture, achieving better feature extraction with lower computational cost compared to the aforementioned hybrid models.	-

**Table 2:** Network Layer Architecture of CTANet.

Layer Name	Input Size	Output Size	Filters (Channels)
Conv Stem	(512, 512, 3)	(128, 128, 96)	96
Downsample-1	(128, 128, 96)	(64, 64, 192)	192
Downsample-2	(64, 64, 192)	(32, 32, 384)	384
Downsample-3	(32, 32, 384)	(16, 16, 768)	768
ConvNeXt-1	(128, 128, 96)	(128, 128, 96)	96
ConvNeXt-2	(64, 64, 192)	(64, 64, 192)	192
ConvNeXt-3	(32, 32, 384)	(32, 32, 384)	384
ConvNeXt-4	(16, 16, 768)	(16, 16, 768)	768
MSM-AMM-1	(128, 128, 96)	(128, 128, 96)	96
MSM-AMM-2	(64, 64, 192)	(64, 64, 192)	192
MSM-AMM-3	(32, 32, 384)	(32, 32, 384)	384
BHAFormer-1	(128, 128, 96)	(128, 128, 96)	96
BHAFormer-2	(64, 64, 192)	(64, 64, 192)	192
BHAFormer-3	(32, 32, 384)	(32, 32, 384)	384
Upsample-1	(16, 16, 768)	(32, 32, 384)	384
Upsample-2	(32, 32, 384)	(64, 64, 192)	192
Upsample-3	(64, 64, 192)	(128, 128, 96)	96
Upsample-4	(128, 128, 96)	(512, 512, 96)	96
Segment Head	(512, 512, 96)	(512, 512, 2)	2 (Classes)

**Table 3:** Ablation study results on the Inria aerial image labeling dataset.

Method	F1	IoU	Precision	Recall
Baseline	89.81	81.51	91.17	88.49
Baseline+MSM-AMM	90.26	82.25	91.43	89.12
Baseline+BHAFormer	90.37	82.43	91.58	89.19
CTANet (Ours)	90.52	82.68	91.75	89.32

**Table 4:** Ablation study results on the Massachusetts building dataset.

Method	F1	IoU	Precision	Recall
Baseline	85.87	75.24	87.52	84.28
Baseline+MSM-AMM	86.33	75.94	87.58	85.11
Baseline+BHAFormer	86.52	76.24	87.75	85.32
CTANet (Ours)	86.70	76.52	88.20	85.25

**Table 5:** Comparison with SOTA methods on the Inria aerial image labeling dataset.

Methods	FLOPs (G)	F1	IoU	Precision	Recall
Segmenter [37]	126.12	89.33	80.72	90.38	88.3
Swin-Unet [39]	97.69	86.54	76.27	86.87	86.21
TransUNet [41]	168.92	89.36	80.77	90.44	88.32
TransFuse [43]	**50.53**	89.32	80.71	90.29	88.38
STT [38]	106.21	87.99	79.42	-	-
BuildFormer [44]	117.12	89.77	81.44	90.75	88.81
CTANet (Ours)	50.77	**90.52**	**82.68**	**91.75**	**89.32**

**Table 6:** Comparison with SOTA methods on the Massachusetts building dataset.

Methods	FLOPs (G)	F1	IoU	Precision	Recall
Segmenter [37]	126.12	84.83	73.65	86.32	83.38
Swin-Unet [39]	97.69	86.35	75.98	86.40	86.31
DC-Swin [40]	72.52	84.12	72.59	83.07	85.19
TransUNet [41]	168.92	85.92	75.31	87.70	84.21
MSRF-Net [6]	81.92	83.90	72.27	87.04	80.98
STT [38]	106.21	85.40	74.51	86.55	84.27
TCNet [42]	91.88	84.29	76.21	85.17	**86.82**
BuildFormer [44]	117.12	86.19	75.74	87.52	84.90
CTANet (Ours)	**50.77**	**86.70**	**76.52**	**88.20**	85.25

**Table 7:** Comparison with SOTA methods on the WHU building dataset.

Methods	FLOPs (G)	F1	IoU	Precision	Recall
Segmenter [37]	126.12	93.49	87.78	93.65	93.34
Swin-Unet [39]	97.69	94.46	89.50	94.53	94.38
DC-Swin [40]	72.52	93.96	88.61	94.38	93.54
TransUNet [41]	168.92	93.57	87.91	93.45	93.69
MSRF-Net [6]	81.92	94.82	90.16	94.97	94.68
STT [38]	106.21	94.97	90.48	-	-
TCNet [42]	91.88	93.95	91.16	95.15	95.55
BuildFormer [44]	117.12	95.53	91.44	95.65	95.40
CTANet (Ours)	**50.77**	**95.74**	**91.84**	**95.86**	**95.63**

**Table 8:** Test results for different transformers in CTANet.

Method	F1	IoU	FLOPs (G)	Params (M)
remove BHAFormer	90.26	82.25	45.9682	37.0048
replace with PVT [33]	90.32	82.35	53.4928	41.5221
replace with Shunted Transformer [35]	90.38	82.45	56.2157	41.9732
replace with DSAFormer [36]	90.36	82.41	51.2760	39.3206
CTANet (Ours)	90.52	82.68	50.7737	39.0978

**Table 9:** The impact of encoder-decoder feature interaction and fusion.

Method	F1	IoU
remove MSM-AMM	90.37	82.43
replace with Skip Connect	90.45	82.56
CTANet (Ours)	90.52	82.68

## Data Availability

Data associated with this research are available online. The Inria dataset is available for download at https://project.inria.fr/aerialimagelabeling/, accessed on 1 January 2024. The Massachusetts dataset is available for download at https://www.cs.toronto.edu/~vmnih/data/, accessed on 4 January 2024. The WHU dataset is available for download at http://gpcv.whu.edu.cn/data/, accessed on 4 January 2024. The code is available at https://github.com/1138256398/CTANet, accessed on 25 September 2024.
